# Internet Derived Information Obstruction Treatment (IDIOT) Syndrome: A Breviloquent Review

**DOI:** 10.7759/cureus.27945

**Published:** 2022-08-12

**Authors:** Karthik Rajaram Mohan, Saramma Mathew Fenn, Ravikumar Pethagounder Thangavelu

**Affiliations:** 1 Oral Medicine, Vinayaka Mission's Sankarachariyar Dental College, Vinayaka Mission's Research Foundation, Salem, IND; 2 Oral Medicine and Radiology, Vinayaka Mission's Sankarachariyar Dental College, Vinayaka Mission's Research Foundation, Salem, IND

**Keywords:** obsessive compulsive disorders, internet, reassurance, domain, cybernorphia

## Abstract

The rapid increase in internet use in the current digital era has caused a potential increase in anxiety, and a person either self-medicates or abruptly stops the drug for his medical illness, thereby the rise in the Internet Derived information Obstructing Treatment (IDIOT) syndrome. The Internet Derived Information Obstructing Treatment (IDIOT) syndrome occurs when patients abruptly quit their treatment because they have blindly trusted internet medical information. WHO calls this an " Infodemic," which has created a complex situation in healthcare, as it has caused too much information in digital and physical environments during an outbreak of the disease and caused mistrust in health authorities. Patients get important health information online and anticipate additional assistance, Dr. C. N. Manjunath, Director, Jayadeva Hospital, says “Doctors are becoming sick because of stress, and they need to take care of their health. There is an added demand to ac­q­u­ire communication skills rather than merely technical and professional qualities.” Patients must not merely believe healthcare-related information available from online health resources and must seek the help of licensed healthcare professionals for their health concerns. There must be an increase in the awareness programs among the public to as not to fall as victims and blindly follow or abruptly stop their medical prescriptions for their medical illness from available online health resources.

## Introduction and background

The current digital era, where everyone has easy access to a smartphone, laptop, or tablet, caused a fulminant increase in the rise of Internet Derived Obstructing Treatment (IDIOT) syndrome. Internet Derived Information Obstructing Treatment (IDIOT) syndrome occurs when patients abruptly quit their treatment because they have blindly trusted internet medical information. Patients get important health information online and anticipate additional assistance. “Doctors are becoming sick because of stress, and they need to take care of their health. There is an added demand to ac­q­u­ire communication skills rather than merely technical and professional qualities” [1]. The WHO defines this infodemic as "too much information during a disease outbreak, including inaccurate or misleading information in digital and physical surroundings." It leads to uncertainty and risk-taking behaviors that are harmful to health.

**Figure 1:**
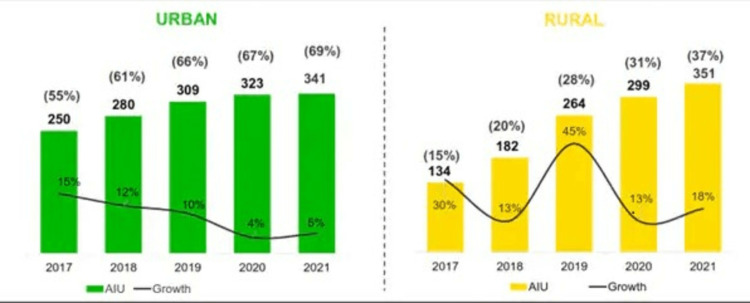
Rural India Accounts for More Than Half the Internet Users Image credit: IAMAI-Kantar. Accessed 1st August 2022. Available from website: https://www.cnbctv18.com/technology/iamai-kantar-report-says-rural-india-accounts-for-more-than-half-the-internet-users-in-country-14283262.htm

Additionally, it diminishes the public health response and fosters mistrust of health officials. Both advantages and disadvantages come with using the internet for medical information. The advantage of using the internet for medical information is that it can facilitate informed conversations with medical professionals and enable the early detection and treatment of dangerous conditions. However, some people also have the chance to stop their drug for their medical illness due to the increased anxiety it causes, thereby making it possible for them to fail and seriously jeopardize the patient's health. Many people self-diagnose their conditions after conducting online research and treating themselves before seeing a doctor. According to the IAMAI (Internet and Mobile Association of India) and data analytics firm Kantar ICUBE 2020 research, the number of active internet users in India is projected to grow from its current level of 692 million users (351 million users from rural India and 341 million users from urban India) in 2021 to 900 million in 2025, an increase of 45% [2] (Figure 1).

## Review

Cyberchondria's etymology

The terms "cyberchondria" and "hypochondria" are related. A cyber is an electronic device, computer network, or usage of a computer for internet communication. Hypochondria is a disorder wherein a person is excessively or unreasonably fearful about contracting a specific illness. Hence, cyberchondria is a computer-related and other phobias related to the internet's use, most likely brought on by it [1].

Triggering or predisposing factors for cyberchondria

People suffering from cyberchondria will experience stimuli from a variety of perspectives, from single traumatic incidents and long-term, untreated stress [2].

Signs of the IDIOT syndrome

**Figure 2:**
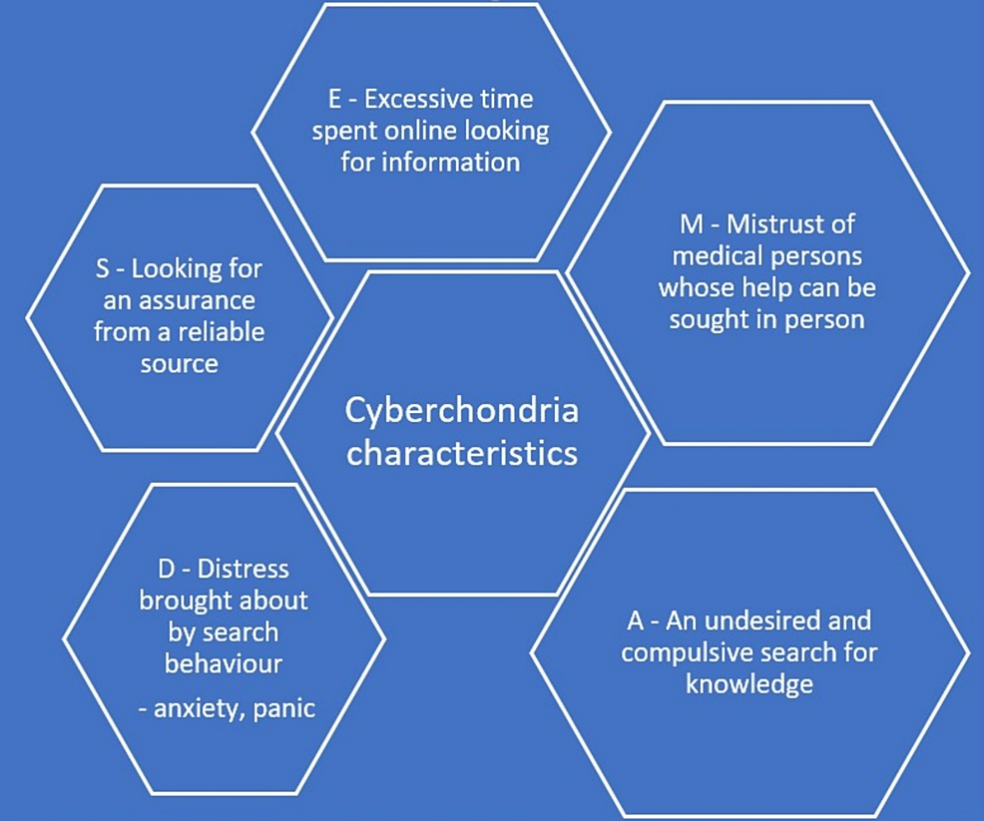
Characteristics of cyberchondria

The main characteristics of cyberchondria include the individual's uncertainty about having a serious illness despite having few or no (EMADS) symptoms, which includes the following: E: Excessive time spent online looking for information. M: Mistrust of medical experts whose opinion could be sought in person. A: An undesired and compulsive search for knowledge. D: Distress brought on by the search behavior, such as anxiety and panic. S: Looking for assurance from a reliable source or person [3] (Figure 2).

On the internet, there is a variety of accurate and reliable information about health and wellbeing, and information on any other subject on the internet is the same in this regard. Some websites post fictitious health information merely to attract users, sponsors, and ratings. People should be wary of websites that build hype while endangering their readers. Such websites ought to be avoided. Numerous trustworthy websites offer clear, accurate fundamental medical information that eventually directs their visitors to seek expert medical assistance. Additionally, there are numerous instances where readers have changed their naive perspective about their health thanks to online medical literature. This has made a significant difference in preventing health issues [3].

Theoretical perspectives of cyberchondria

Initial investigations into cyberchondria were guided by the reassurance-seeking theory, which proposes that people with high levels of health anxiety engage in Online Health Resources (OHR) to feel better about their health worries. Since the internet is inherently unexpected, some people are encouraged by what they see online while others are not [4].

Someone who doesn't feel reassured or feels only partially reassured continues using online health resources to feel reassured. This makes them more apprehensive. Online Health Resources (OHR) thrives due to the factors that support it, such as the need for clear-cut explanations, internet addiction, the uncertainty of an online search technique for a medical procedure, and the dubious reliability of online information sources. Cyberchondria is viewed as a problematic perpetuating mechanism for health concerns that initiates a constant, vicious cycle. This model is consistent with the phenomenon later referred to as "problematic Online Health Resources" [4].

The metacognitive theory of cyberchondria states that certain metacognitive beliefs are connected to the search for information through online health resources and cause detrimental effects, such as increased suffering and anxiety. These views could be about the Internet's utility in handling information or resources from the internet (a positive metacognitive effect), or they could be destructive due to increased suffering and anxiety it causes (negative metacognitive effect). The pattern is known as "compulsive Online Health Resources." It is characterized by perceiving Online Health Resources as stressful, monomaniacal, and out of control if the negative metacognitive beliefs are more prominent. Compulsive Online Health Resources and cyberchondria linked to negative metacognitive beliefs may have a stronger relationship with problematic internet use (PIU), while problematic Online Health Resources and cyberchondria based on reassurance seeking is more obviously and strongly related to health anxiety. As a result, dysfunctional OHR and compulsive OHR could be considered "pathological OHR" [5,6].

The two types of pathological OHR, and hence the two "subtypes" of cyberchondria, need to be further theorized and tested. The consequences for understanding, preventing and regulating cyberchondria and the degree to which these two patterns are distinct remain unclear. Although both obsessive and problematic OHR may exist to differing degrees in different persons and sometimes at different times in the same person, it has been suggested that cyberchondria may be a "design-build" that embraces both. If this is the case, we need to develop a comprehensive formulation of cyberchondria that specifies the possible mechanisms and reinforcing variables [7,8].

Another crucial issue is cyberchondria's conceptual independence. Cyberchondria may be relatively particular and different from all linked constructs, as revealed by the network analysis study, as was previously mentioned. Before cyberchondria can be conceived as an entity in and of itself, this result needs to be replicated-a challenging effort given the lack of clear rules about the minimal prerequisites for any psychopathological entity to be deemed separate and different from associated illnesses and health issues. These factors could aid in more effectively integrating the varying factors that cause cyberchondria [9].

Strategies to combat cyberchondria

Stay away from shaming oneself, be compassionate with yourself. Practice relaxation techniques like deep breathing exercises. Resisting the notion of being ill. Communicate with a medical professional. Understanding the broader, clear, context picture [10].

The various domains of WHO-QOL are presented in Table 1.

**Table 1:** WHO-QOL Domains

Domain I Physical	Domain II Psychological	Domain III Level of independence	Domain IV Social relationships	Domain V Environment	Domain VI Spirituality/ Religion
1. Pain and Discomfort	6.Positive feelings	11. Mobility	17. Personal relationships	20. Freedom, physical safety and security	29. Overall quality of life and general health perceptions
2. Energy and Fatigue	7.Thinking, learning, memory, and concentration	12.Activities of daily living	18. social support	21. Home environment	
3. sexual activity	8.Self-esteem	13.Dependance on medicinal substances and medical aids	19. Activities as provider/supporter	22. Work satisfaction	
4. Sleep and rest	9.Bodily image and appearance	14.Dependence on non-medicinal substances (alcohol, tobacco, drugs)		23. Financial resources	
5. sensory functions	10.Negative feelings	15. Communication capacity		24. Health and social care: Accessibility and quality	
		16.Work capacity		25. Opportunities for acquiring new information and skills	
				26. Participation in and opportunities for recreation and leisure activities	
				27. Physical environment (Pollution/noise/traffic/climate)	
				28. Transport	

Severity scales used to measure cyberchondria

The severity of cyberchondria can be assessed with the help of the following questionnaire scales as cyberchondria severity scale (CSS), WHO-QOL, WHO-QOL-100, and WHO-BRET [11]. 

Cyberchondria Severity Scale (CSS)

McElroy and Shevlin developed the Cyberchondria Severity Scale (CSS) in 2014 to assess how internet searches for medical health-related information influence a person's decisions and learning. Repetitive and excessive internet searches for medical health-related pieces of information due to easy accessibility (excessiveness), behavioral interference in day-to-day life activities; increased time spent for online searches due to addiction (compulsiveness), increased negative affect when engaging in excessive searches such as increased suffering caused by anxiety or fear (distress), and repeated attempts to seek reassurance from medical health professionals based on information obtained through online searches are the four dimensions of cyberchondria, as determined by factor analysis studies of the CSS (reassurance). The CSS is a self-report comprising 33 questionnaire items that measure anxiety and health-related online search behavior, based on a five-point Likert scale ranging from never to always to indicate the extent to which each statement applies to their daily experience (for example, "I feel more relaxed after looking up symptoms or alleged medical issues online, afraid, or distressed") [10].

CSS-PL

The CSS-PL is a preliminary standardized version of a cyberchondria measurement tool that satisfies the psychometric requirements for validity and reliability in psychological testing tools. The CSS-PL can be applied to scientific research and diagnostics [11].

WHO-QOL -100

How people feel about themselves in relation to their goals, aspirations, standards, fears, and the culture and value systems in which they were nurtured is measured by the WHOQOL-100. It is a 100-question test that is now available in 29 language variations in directly comparable forms. It produces a multi-dimensional profile of quality of life ratings across domains and sub-domains (facets) [11].

WHO-BREF

The WHO-BREF comprises 24 questionnaires taken from four domains: physical health (seven items), social relationships (three items), psychological health (six items), and environmental health (eight items). The WHOQOL-BREF will allow medical professionals to evaluate how well patients are doing after therapy [12].

Bhaumik U and Nayok S (2021) stated that cyberchondria is one of the emerging threats in the COVID-19 pandemic [13].

*Treatment modalities for IDIOT syndrome:* Researchers have used the term for the acknowledged condition known as "health anxiety." The following therapy modalities are successful in treating health anxiety [14,15]

*The use of mindfulness in cognitive therapy (MBCT)*: For individuals with health anxiety, mindfulness-based cognitive therapy (MBCT) is a helpful supplement to "normal services." Patients who participated in MBCT and received customary or so-called services demonstrated much lower health anxiety than those who did not. This improvement was noticeable immediately the following therapy and at a year-long follow-up evaluation [16].

*Therapy for rational emotive behavior*: Learning how to react correctly to neutral or unclear messages from one's body is a skill that can be learned with the help of rational emotive behavior therapy. The goal of teaching clients distraction strategies is to get them to shift their attention away from their health anxiety or symptoms. They also learn relaxation techniques to control their anxiety and its 11 bodily symptoms [17-19].

*Pharmacotherapy*: Drugs like selective serotonin reuptake inhibitors (SSRIs) have effectively treated the obsessive thoughts associated with anxiety and cyberchondria. The similarities between OCD and cyberchondria/illness anxiety disorder serve as the foundation for this therapy. The obsessive and compulsive characteristics of 12 cyberchondria may be lessened by the same drugs that are effective in treating OCD [20-23].

## Conclusions

In the case of IDIOT syndrome, there is a growing demand for public education on what, where, and how to look up a diagnosis and a cure online. The expectations of patients and their caregivers have grown as a result of the rapid advancement of technology. When patients rush to the hospital with inflated expectations of the medical staff, the doctors are overburdened. The demand for doctors and nurses to work nonstop is getting worse every day. Given the burden on doctors, they need to take care of their own health, and many have had challenges with their health and are getting sick due to stress. There is a greater lack of trained workers because many people have become unwell and have not returned to work. Don't fall victim to the IDIOT syndrome is our counsel. An increase in the awareness of IDIOT syndrome is essential among patients who take self-medication, as medicines are accessible from nearby pharmacies by searching medical information from online health resources. People must refrain from searching for medical information through the internet and must seek the help of licensed health care professionals for their medical health problems. 
